# Master Transcription Regulators and Transcription Factors Regulate Immune-Associated Differences Between Patients of African and European Ancestry With Colorectal Cancer

**DOI:** 10.1016/j.gastha.2022.01.004

**Published:** 2022-03-30

**Authors:** Parvathi A. Myer, Hyunjin Kim, Anna M. Blümel, Ellen Finnegan, Alexander Kel, Taylor V. Thompson, John M. Greally, Jochen HM. Prehn, Darran P. O’Connor, Richard A. Friedman, Aris Floratos, Sudipto Das

**Affiliations:** 1 Montefiore Medical Center, Albert Einstein Cancer Center, Bronx, NY; 2 St. Jude’s Children’s Research Hospital, Memphis, Tennessee TN; 3 School of Pharmacy and Biomolecular Sciences, Royal College of Surgeons, Dublin, Ireland; 4 Department of Physiology and Medical Physics, Royal College of Surgeons in Ireland, Dublin, Ireland; 5 GeneXplain GmbH, Wolfenbuettel, Germany; 6 BIOSOFT.RU, LLC, Novobirsk, Russia; 7 Institute of Chemical Biology and Fundamental Medicine SBRAS, Novobirsk, Russia; 8 Albert Einstein College of Medicine, Bronx, NY; 9 Biomedical Informatics Shared Resource, Herbert Irving Comprehensive Cancer Center, and Department of Biomedical Informatics, Columbia University Irving Medical Center, New York, NY; 10 Department of Systems Biology, Columbia University, New York, NY; 11 Department of Biomedical Informatics, Columbia University, New York, NY

**Keywords:** Health Disparities, Colorectal Cancer, Genomic Profiling, African Americans.

## Abstract

**Background and Aims:**

Individuals of African (AFR) ancestry have a higher incidence of colorectal cancer (CRC) than those of European (EUR) ancestry and exhibit significant health disparities. Previous studies have noted differences in the tumor microenvironment between AFR and EUR patients with CRC. However, the molecular regulatory processes that underpin these immune differences remain largely unknown.

**Methods:**

Multiomics analysis was carried out for 55 AFR and 456 EUR patients with microsatellite-stable CRC using The Cancer Genome Atlas. We evaluated the tumor microenvironment by using gene expression and methylation data, transcription factor, and master transcriptional regulator analysis to identify the cell signaling pathways mediating the observed phenotypic differences.

**Results:**

We demonstrate that downregulated genes in AFR patients with CRC showed enrichment for canonical pathways, including chemokine signaling. Moreover, evaluation of the tumor microenvironment showed that cytotoxic lymphocytes and neutrophil cell populations are significantly decreased in AFR compared with EUR patients, suggesting AFR patients have an attenuated immune response. We further demonstrate that molecules called “master transcriptional regulators” (MTRs) play a critical role in regulating the expression of genes impacting key immune processes through an intricate signal transduction network mediated by disease-associated transcription factors (TFs). Furthermore, a core set of these MTRs and TFs showed a positive correlation with levels of cytotoxic lymphocytes and neutrophils across both AFR and EUR patients with CRC, thus suggesting their role in driving the immune infiltrate differences between the two ancestral groups.

**Conclusion:**

Our study provides an insight into the intricate regulatory landscape of MTRs and TFs that orchestrate the differences in the tumor microenvironment between patients with CRC of AFR and EUR ancestry.

## Introduction

African Americans have the highest incidence and mortality from colorectal cancer (CRC) compared with other ethnic groups.^1^ Moreover, African Americans present with CRC at an earlier age with more frequent proximal CRCs.^1^ Social determinants of health such as low income, neighborhood racial segregation, and lower education levels have been associated with reduced rates of colon cancer screening, resulting in higher incidence of CRC in African Americans.^2^ Although differences in these social determinants of health and increased rates of obesity, decreased physical activity, and access to health care explain some of the observed health disparities, the molecular events that underpin differences in CRC tumor biology between patients of African (AFR) and European (EUR) ancestry have not been fully explored.^3^, ^4^, ^5^, ^6^ This is partially explained by the fact that prior large-scale studies performing population-based genomic sequencing had limited representation of African Americans.^3^^,^^6^, ^7^, ^8^ Addressing these gaps has become particularly important, following reports that show substantial differences in the genome, transcriptome, and epigenome between patients with cancer from these two distinct ancestry groups.^9^

It is well understood that disease etiology and presentation vary greatly between ethnic groups, with mutation and gene expression patterns likely regulating phenotypic variations across cancer types.^10^^,^^11^ One such phenotypic difference involves differences in the tumor microenvironments of colorectal,^12^ breast,^13^ and prostate cancer.^14^ This phenotypic disparity is underpinned by the significant differences in immune cell infiltrates between patients with cancer of AFR and EUR ancestry. Specifically, recent reports show that African American patients with CRC have lower levels of cytotoxic T lymphocytes and higher numbers of exhausted CD8+ cells relative to Whites with CRC.^12^^,^^15^ Although the differences in immune profiles between the two ancestry groups are becoming apparent, the precise molecular processes that regulate these immune differences remain unclear.

The tumor microenvironment is inherently complex and dynamic. This is reflected in the gene expression patterns that are dependent on signaling pathways, transcriptional regulators, and epigenetic processes. A growing body of evidence suggests that rewiring of signal transduction pathways is regulated by molecules termed master transcriptional regulators (MTRs), which are represented at the top of the signal transduction hierarchy. They are reported to modulate gene expression through key transcription factors (TFs), usually by a positive feedback loop.^16^^,^^17^ Given the important regulatory role of these MTRs and TFs, they may control underlying immune-associated gene expression changes and consequentially affect overall tumor microenvironment differences between patients of AFR and EUR ancestry. Similarly, epigenetic alterations such as DNA methylation are not only influenced by genotype and cell subtype composition; they may also reflect cellular reprogramming influenced by life style differences and environmental factors. By studying an epigenetic regulator like DNA methylation, we may infer insights into the observed phenotypic differences between AFR and EUR patients with CRC, including differences in their tumor microenvironment.^18^

Although previous multiomics studies have shown that widespread genetic, epigenetic, and transcriptomic changes probably contribute toward CRC pathogenesis, the impact of these alterations, particularly in regulating the observed immune cell differences between the patients with CRC of AFR and EUR ancestries, still remains to be understood. Our study aimed to identify the regulatory factors potentially mediating the differences in immune cell composition in the tumor microenvironment between AFR and EUR patients with CRC. We used a comprehensive integrative analysis of somatic mutations, gene expression, DNA methylation, and immune cell type abundances by analyzing data from The Cancer Genome Atlas (TCGA).

## Methods

### Ancestry determination and demographic analysis

The genotypic ancestry of all 636 patients with CRC from the TCGA was determined through co-clustering with a population reference panel from the 1000 Genomes Project. The genotype data of the 1000 Genomes Project participants and those of the TCGA patients were combined together and used for constructing a principle component analysis using EIGENSTRAT. Using principal component analysis, the samples were clustered based on self-reported race. We calculated the Euclidean distance between the mean values of the clusters of the 1000 Genomes Project genotypes and those from each TCGA patient based on PC1, PC2, and PC3. The shortest distance for each TCGA patient to the races defined by the 1000 Genomes Project clusters was used to define the test sample’s predicted race.

After ancestry determination, we carried out detailed demographic analysis for all 609 patients with CRC with predicted AFR and EUR ancestry using various clinical variables available for these patients, including microsatellite status: high (MSI-H), low (MSI-L), and stable (MSS) patients, body mass index, age, and location and stage of disease as obtained from the TCGA database. Cross-tabulation of both population characteristics was carried out using SPSS. The chi-squared test was used to obtain *P*-values with a significance threshold of ≤0.05. *P*-values that were adjusted for multiple testing (where applicable) using the Benjamini-Hochberg method are represented as “*q-values*”, and unadjusted *P*-values are represented as “*P-values*”. Unknown or missing values were omitted from the analysis. Given that as usable multiomics (somatic mutation, RNA sequencing, and DNA methylation data) was only available for 55 AFR and 456 EUR ancestry patients with MSS (includes microsatellite low and stable) CRC, these patients were used for further downstream analysis including whole-exome, RNA sequencing, and DNA methylation analysis (Figure A1B).

### Whole-exome sequencing analysis

We downloaded the mutation annotation format (MAF files) generated by the TCGA project using four different pipelines: MuSe, MuTect2, SomaticSniper, and VarScan2. For each sample, we combined the output of all callers (ie, variants were retained as long as they were called by at least one variant calling pipeline). The patients were grouped using various clinical parameters, including age, location of tumor, and ancestry, resulting in a total of 42 groups. Specifically, Variant call files (MAF files) generated using MuSe, MuTect2, SomaticSniper, and VarScan2 were combined for each sample, ensuring retention of a variant called by at least one variant calling pipeline. POLE-mutated samples were subsequently removed, and MSI-L samples were combined with the MSS group. The patients were grouped using various clinical parameters, including age, location of tumor, and ancestry, resulting in a total of 42 groups. For each group, the somatic mutation summary plot was generated along with computing driver genes followed by pathways analysis of the driver genes. Subsequently, tumor mutation burden (TMB) was determined between MSS-AFR vs MSS-EUR. For this purpose, TMB was computed by counting all mutations in the MAF files. The Oncodrive CLUST^19^ method was used for identifying the driver genes in each group. This methodology is based on the concept of clustering of mutations in certain hotspots of the genome, with a cluster score of 1 signifying a single hotspot encompasses all observed variants. The TMB calculation and driver gene extraction were carried out with the *maftools* (v2.0.15) package. For each group, a somatic mutation summary plot (*ggplot2* (v3.1.0) and *ggrepel* (v0.8.0)) was generated along with computing driver genes and pathway analyses (*clusterProfiler* (v3.10.0)) of these genes.

### RNA sequencing and MCP-counter analyses

For the RNA sequencing data analysis, we excluded formalin fixed paraffin embedded-derived patient samples. For multiple samples for one patient, the one with the highest RNA integrity number was chosen. For multiple transcripts mapping to the same gene, all relevant transcript read counts were added up to generate an aggregate read count for the gene. Genes with effective zero expression (ie, that have N/A, 0, or 1 across reads across all samples) are removed, to improve statistics in downstream analyses. The differential gene expression analysis was performed using the *DESeq2* package (v1.22.1). Volcano plots were generated based on the differentially expressed genes (DEGs) with the false discovery rate (FDR) <0.05 of each comparison using *ggplot2* package (v3.1.0). Next, given that as we find age and tumor location to be significantly different between AFR and EUR patients with CRC, we corrected all DEGs for age and tumor location by including these factors as covariates in the *DESeq2* linear model. Finally, gene ontology was carried out using the DAVID functional annotation tool for molecular function.

For the microenvironment cell population–counter (MCP-counter) analysis, POLE-mutated samples were removed, and all the samples were normalized with variance-stabilizing transformation. Cell frequencies were then computed for all samples, using the *MCPcounter* package (v1.1.0) in R.^20^ For each comparative analysis, we applied the Wilcoxon signed-rank test to compare immune cell estimates between patients of AFR vs EUR ancestry, young vs old, stage I vs II vs III vs IV, and location—right- vs left-sided tumor.

### Transcription factor binding analysis

TF binding sites (TFBSs) in promoters and enhancers of DEGs (corrected for age and tumor location as mentioned in the previous section) were analyzed using known DNA-binding motifs described in the TRANSFAC library,^21^ release 2020.2 (geneXplain GmbH, Wolfenbuttel, Germany). For each gene, we searched for so-called “composite modules”, defined as clusters of different TFBSs that act as potential condition-specific enhancers—in their surrounding regulatory regions (from -1000 bp upstream to +100 bp downstream of the transcription start site [TSS]) and identified TFs predicted to regulate activity of the genes. We applied the Composite Module Analyst (CMA) method^22^ to characterize these potential enhancers, as targets of multiple TFs bound in a cooperative manner to the regulatory regions of the genes of interest. Specifically, CMA applies a genetic algorithm to construct a generalized model of the enhancers by specifying combinations of TF motifs (from TRANSFAC) whose sites are most frequently clustered together in the regulatory regions of the studied genes.^23^ The enrichment was computed using CMA software^22^ incorporated in the geneXplain platform (genexplain.com/). In the second step of the TSS analysis, common regulators of the candidate TFs were identified. These “MTRs” are major candidates for therapeutic targeting as they are the ultimate regulators of intracellular pathways that activate the pathological processes under investigation. The MTR search uses the TRANSPATH database,^24^ release 2020.2 (geneXplain GmbH, Wolfenbuttel, Germany). A comprehensive signal transduction network of human cells was built by the software on the basis of reactions annotated in TRANSPATH.

### DNA methylation analysis

Beta values derived from Illumina 450K microarrays were used for each patient for the DNA methylation analysis. Data preprocessing involved removal of duplicated and POLE-mutated samples. This was followed by removal of probes with uninformative “N/A” values, cross-reactive probes, probes on sex chromosomes, all non-CpG as well as SNP-related probes, and multihit probes using the Chip Analysis Methylation Pipeline (*ChAMP*) package (v2.12.4). Next, normalization of the beta values was performed using beta-mixture quantile normalization. Subsequently, differentially methylated CpG positions (DMPs) were identified using the *ChAMP* package and mapped to individual probes on the microarray. To this end, we identified DMPs between normal tissue and tumor for both AFR and EUR patients with CRC, enabling us to generate a list of DMPs that are tumor specific. These tumor-specific DMPs identified for both AFR and EUR patients were further compared with each other to identify DMPs between patients with CRC of AFR and EUR ancestry. Next, similar to the RNA sequencing analysis, the DMPs were corrected for both age and tumor location by including these factors as covariates in the *ChAMP* linear model. A heatmap of the beta methylation values corresponding to these DMPs was generated using the *ComplexHeatmap* package v2.4.2. on R. Annotation of patients into DNA methylation clusters was performed using the *HeatmapAnnotation* function that was also available from this package.

We first associated the DMPs to genes located within 3’/5′ untranslated region, gene body, intergenic enhancer regions, and promoters (−1200 bp to +200 bp relative to the TSS) of the DMP. Next, we generated a correlation plot—methylation vs expression values for every DEG (FDR <0.05) and associated DMPs. In case where genes associated with multiple DMPs, the CpG with the lowest FDR was used. This resultant correlation plot allowed us to identify “methylation-sensitive gene”, which showed a significant negative correlation (*P* < .05) between methylation and gene expression.

## Results

### Defining ancestry of patients with CRC and characteristics of disease

Given that information on race (self-reported) was available only for 374 of 636 patients with CRC available from the TCGA database, we carried out ancestry prediction using data from the 1000 Genomes Project. Our ancestry caller classified >95% of patients with CRC with missing self-reported race information (Figure A1A, 1B). When race was recorded in the clinical data, we observed a >98% concordance between self-reported and computationally inferred ancestry. We next carried out demographic analysis of CRC in both AFR and EUR patients with predicted ancestry including both MSS and MSI-H patients, which confirmed from prior epidemiologic studies the increased prevalence of early-age onset (<50 years) CRC in AFR compared with EUR patients (*P* = .009)^25^ and predominantly right-sided tumors in AFR (*P* = .001).^1^ In addition, the frequency of MSI-H tumors was higher in EUR than that in AFR (14.3% vs 11.8%, respectively; *P* = .032 (Table)). Ultimately, among patients with MSS CRC and with predicted AFR and EUR ancestry, we identified a total of 55 AFR and 456 EUR patients for whom usable multiomics data were available and, therefore, were used for all downstream analyses (Figure A1B).

**Table:** Demographic Analysis of Patients With CRC and With Predicted African (AFR) and European (EUR) Ancestry

Characteristic	Total		Africans		Europeans		*P*-value
	N	%	N	%	N	%	
Total	609	100.0	65	10.7	544	89.3	
Age at diagnosis							**0.009**
30–39 y	16	2.6	3	4.6	13	2.4	
40–49 y	57	9.4	10	15.4	47	8.6	
50–59 y	97	15.9	18	27.7	79	14.5	
60–69 y	171	28.1	18	27.7	153	28.1	
70–79 y	167	27.4	8	12.3	159	29.2	
≥80 y	98	16.1	7	10.8	91	16.7	
Missing	3	0.5	2	3.1	1	0.2	
Mean, y (SD)	66	(12.8)	60.3	(13.6)	67	(12.5)	
Sex							**0.378**
Female	286	47.0	34	52.3	252	46.3	
Male	320	52.5	30	46.2	290	53.3	
Missing	3	0.5	2	3.1	1	0.2	
Body mass index							**0.032**
Underweight^a^	5	0.8	1	1.5	4	0.7	
Normoweight^b^	84	13.8	14	21.5	70	12.9	
Overweight^c^	118	19.4	18	27.7	100	18.4	
Obese, Class I^d^	49	8.0	9	13.8	40	7.4	
Obese, Class II^e^	38	6.2	15	23.1	23	4.2	
Missing	315	51.7	8	12.3	307	56.4	
Mean kg/m^2^ (SD)	28.3	(6.3)	30.5	(7.7)	27.7	(5.8)	
Tumor stage							**0.428**
I	105	17.2	9	13.8	96	17.6	
II	215	35.3	20	30.8	195	35.8	
III	176	28.9	22	33.8	154	28.3	
IV	90	14.8	13	20.0	77	14.2	
Missing	23	3.8	2	3.1	21	3.9	
Tumor site							**0.001**
Right-sided colon	211	34.6	34	50.8	178	32.7	
Left-sided colon	179	29.4	19	27.7	161	29.6	
Rectosigmoid junction and rectum	159	26.1	4	6.2	155	28.5	
Transverse colon	36	5.9	7	10.8	29	5.3	
Missing	24	3.9	4	6.2	20	3.7	
Microsatellite instability status							**0.032**
Indeterminate	3	0.5	2	3.1	1	0.2	
MSI-H	83	14.0	8	12.3	75	13.8	
MSI-L	99	16.3	10	15.4	89	16.4	
MSS	423	69.5	46	70.8	377	69.3	
Missing	1	0.2	0	0.0	1	0.2	

The bold entries represent the *P*-values calculated for each clinical characteristic for CRC patients of African and European ancestry calculated using the chi-squared test with a significance threshold of *P* < 0.05. SD, standard deviation.

a Underweight [<18.5 kg/m^2^].

b Normal weight [18.5–24.99 kg/m^2^].

c Overweight [25–29.99 kg/m^2^].

d Obese, Class I [30–34.99 kg/m^2^].

e Obese, Class II [≥35 kg/m^2^].

To this end, contrary to literature, exome sequencing data analysis showed that there were no significant differences in overall survival (segregated by stage) and TMB between AFR and EUR patients (Figure A1C and D). Previous studies using sequencing of gene panels have shown that African Americans have more frequent *KRAS* mutations and less frequent *BRAF* mutations.^26^^,^^27^ Our data show that although there were no statistically significant somatic mutational differences or overall mutation burden by ancestry, AFR patients tended to have more frequent *KRAS* and *PIK3CA* mutations (Figure A1E).

### Gene expression analysis reveals a significant downregulation of immune-associated pathways and reduced cytotoxic lymphocyte and neutrophils in AFR patients with CRC

**Figure 1:**
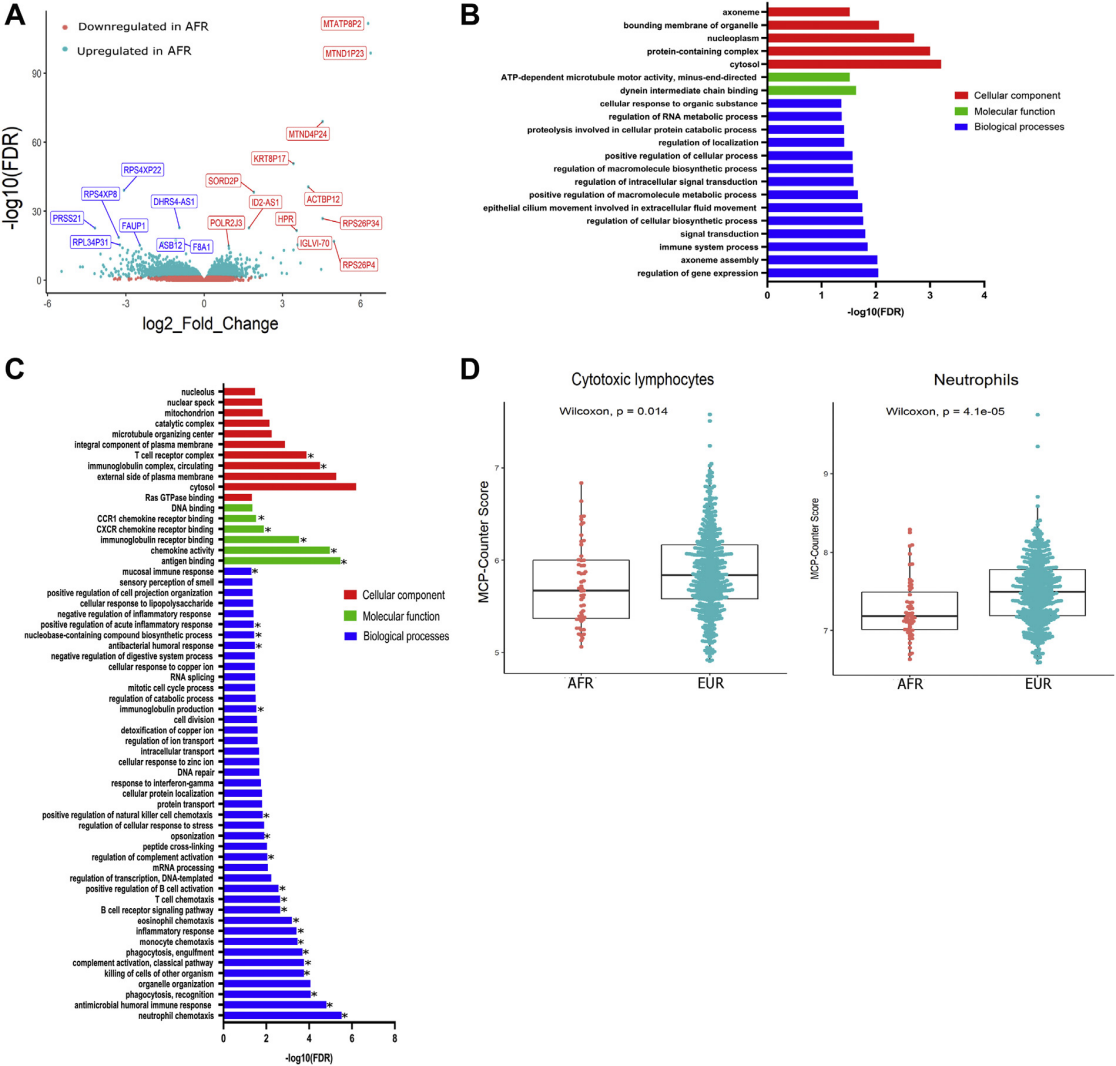
Gene expression and tumor immune infiltrate differences in African and European ancestry patients with CRC. (A) The volcano plot is based on DEGs. The x-axis is “log_2_FoldChange”, and the y-axis is -log_10_(padj). The log_2_FoldChange is the logarithm form of the fold change between the AFR and EUR patients. The gene names of top 10 upregulated and downregulated genes in AFR patients relative to the EUR are highlighted (q < 0.05). The bar graph shows the most significantly enriched Gene Ontology (GO) terms for the (B) upregulated and (C) downregulated differentially expressed genes. The y-axis represents all the GO terms (biological processes, molecular function, and cellular component), and the x-axis indicates the log_10_FDR. It is notable that a large proportion of the enriched GO terms in the downregulated genes are associated with vital immune-associated processes (indicated by asterisk). (D) The graphs represent the specific immune cell populations that demonstrate a significant difference in abundance between the AFR and EUR patients with CRC. Applying the RNA sequencing data to MCP-counter–based analysis shows that both cytotoxic lymphocytes (*P* = .014) and neutrophils (*P* = .0004) are significantly lower in AFR vs EUR patients (x-axis) based on their MCP-counter score (y-axis). The *P*-values are calculated using the Wilcoxon signed-rank test.

Gene expression analysis of RNA sequencing data identified 1942 DEGs between the AFR and EUR patients with CRC. Given that the differential gene expression analysis was corrected for age and tumor location (the two clinical covariates significantly different between AFR and EUR patients—Table), the DEGs therefore represented gene expression differences arising purely due to ancestry. Specifically, these included 766 upregulated (>1.5-fold, q < 0.0.5) and 1176 downregulated (<0.5-fold, q < 0.05) genes in AFR relative to EUR patients (Figure 1A, Table A1). Gene ontology analysis identified “regulation of gene expression” and “neutrophil chemotaxis” as the most enriched biological processes in upregulated and downregulated genes, respectively (Figure 1 B,C, Table A2 ). In addition, although pathway enrichment analysis was uninformative for upregulated genes, it did, however, reveal three canonical pathways enriched in downregulated genes including “cytokine-cytokine interaction” (28 genes, q < 0.00005), “chemokine signaling pathway” (17 genes, q = 0.007), and “mineral absorption” (8 genes, *P* = .015) (Table A2). This is consistent with the observation that most downregulated genes are associated with immune regulation (Figure 1B).

We next carried out an immune cell deconvolution by applying RNA-seq data (uncorrected for any clinical covariates) MCP-counter analysis to identify differences in the abundance of immune cell types in patients with CRC of AFR vs EUR ancestry. This analysis showed that both cytotoxic lymphocytes (*P* = .014) and neutrophil cell populations (*P* = .00004) are significantly decreased in AFR compared with EUR patients (Figure 1D, Figure A2). Given that age, location of tumor, and stage are identified as clinical covariates that are different between patients with CRC of AFR and EUR ancestry, it is likely that these covariates impact the observed immune cell differences between AFR and EUR patients as identified from the MCP-counter analysis. To examine further if this influence was indeed the case, we first performed a two-way analysis of variance to ascertain if the impact of ancestry on cytotoxic T lymphocytes and neutrophils is confounded by age, stage, and location. First, this analysis showed that for cytotoxic T lymphocytes, ancestry was consistently a significant predictor of high vs low cytotoxic T lymphocytes when evaluated along with ancestry vs age (*P* = .0366), stage (*P* = .0407), and location (*P* = .00607). In addition, location of the tumor, that is, right vs left, (*P* = .00615) and stage (*P* = .0130) were also significant independent predictors of high vs low cytotoxic T lymphocytes across all the samples of patients with CRC. However, age (young vs old) was not a significant predictor (*P* = .15) of high vs low cytotoxic T lymphocytes (Figure A3). In terms of neutrophils, similar to cytotoxic T lymphocytes, ancestry was again a significant predictor of high vs low neutrophils when evaluated along with ancestry vs age (*P* = .0005), stage (*P* = .0004), and location (*P* = .0044). However, in contrast to cytotoxic T lymphocytes, independent of ancestry, location of the tumor was the only significant predictor (*P* = .00232) of high vs low neutrophils (Figure A3). These results are further supported by our analysis of each covariate across all immune cell types. For instance, when factoring in age (young vs old), we observe that only neutrophils are significantly decreased in the AFR compared with EUR patients (*P* = .00023) (Figure A4). Interestingly, when factoring in tumor location (right vs left colon), both cytotoxic lymphocytes (*P* = .0013) and neutrophils (*P* = .0007) were significantly reduced in AFR patients compared with EUR patients with left-sided CRC. However, although a similar tendency is noted in AFR vs EUR with right-sided CRC tumors, this difference was not statistically significant (Figure A5). This is surprising given that previous reports have demonstrated that left-sided CRC tumors have higher levels of immune infiltration evidenced by higher cytotoxic activity score, T-cell infiltration, and CD8 T-cell or Treg.^28^ Similarly, when accounting for the stage of disease, neutrophils were significantly reduced in AFR patients compared with EUR patients with both patients with stage I (*P* = .0027) and IV (*P* = .0014) CRC. In addition, no significant differences in cytotoxic lymphocytes were observed between AFR and EUR patients across any stage of CRC disease. This is consistent with the finding that advanced stage CRC exhibits an overall lower level of immune infiltrates.^29^ We do, however, observe that in patients with stage I CRC, in addition to neutrophils, other immune cell types such as fibroblasts (*P* = .014) and monocytic lineage cells (*P* = .008) were significantly reduced in AFR patients when compared with EUR patients. Similarly, in patients with stage IV CRC, apart from neutrophils, endothelial (*P* = .042) myeloid dendritic cells (*P* = .016) were significantly reduced in AFR patients when compared with EUR patients (Figure A6). Taken together, these results demonstrate that several immune-mediated processes are largely downregulated in the AFR patients with CRC and reflect the reduced levels of cytotoxic lymphocytes and neutrophil levels in this patient cohort compared with EUR patients.

### Regulation of key immune cell type differences potentially mediated by a complex interplay between MTRs and TFs

Gene expression changes are often orchestrated by regulatory factors including TFs and MTRs. To determine if the observed gene expression and immune cell differences may be regulated by these TFs and MTRs, we carried out an in silico analysis to identify TFBSs and key MTRs enriched within the DEGs corrected for age and location of tumor in patients with CRC with AFR and EUR ancestry.

TFBS analysis identified 287 and 257 TFs whose binding sites were enriched (q < 0.05) in the upregulated and downregulated genes, respectively, in the cohort of AFR patients with CRC (Table A3). Given that TFs often work in a co-operative manner to regulate gene expression, we next looked for composite modules localized near DEGs, that is, sets of TFBSs that tend to form clusters owing to their interaction with each other.^16^ We identified a composite module consisting of binding motifs for 7 TFs (MyoD, Rad21, POU2F1, BCL6, OCT3, c-FOS, and YY1) in upstream regions of downregulated genes. We also identified a composite module of binding motifs for 10 TFs (NFκB, GR, MAFK, PR, E2A, SLUG, LEF-1, EVI-1, AML3, and YY1) in the upstream regions of upregulated genes (Figure A7).

**Figure 2:**
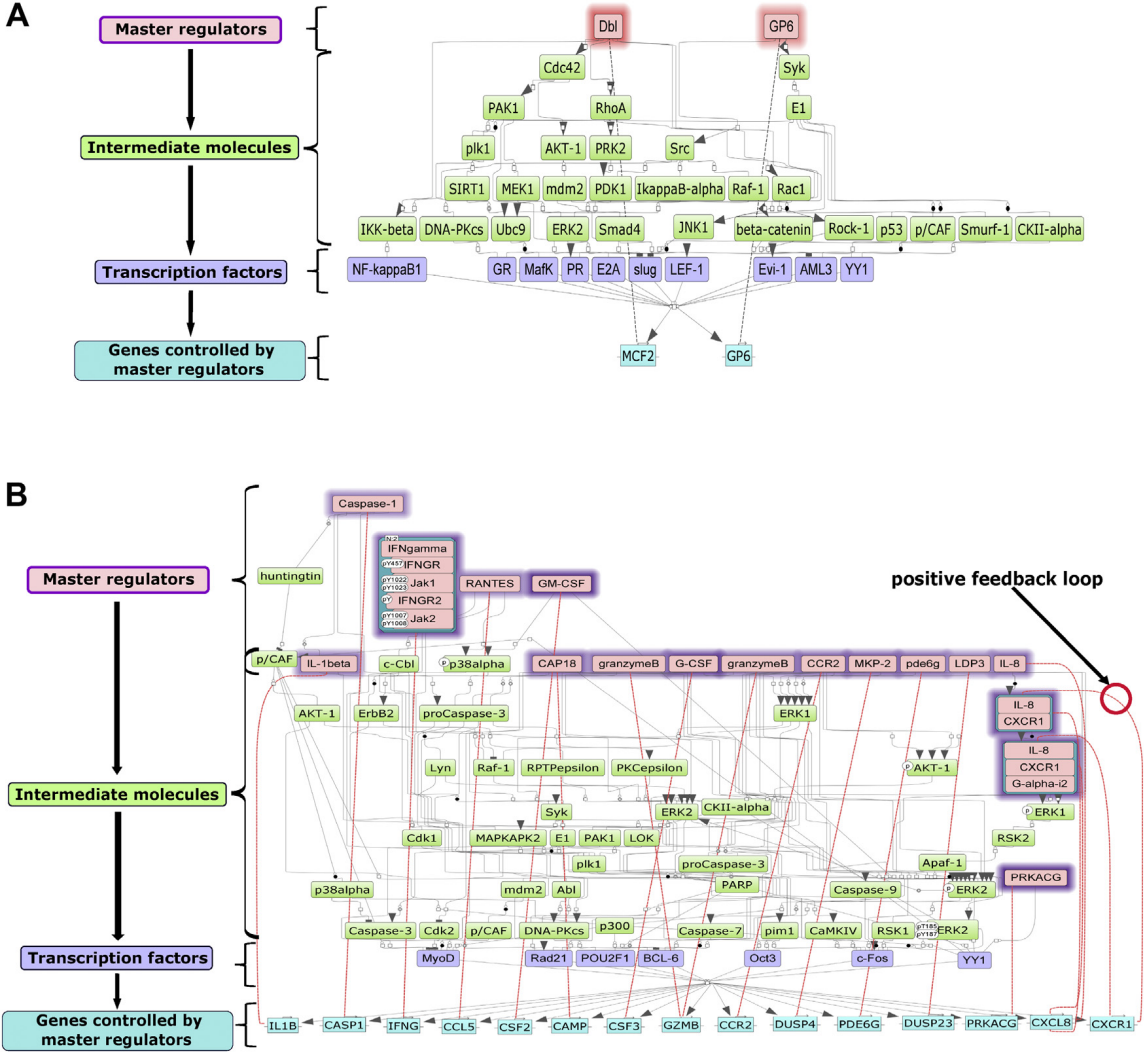
Regulation of gene expression by master transcriptional regulators and transcription factors. The diagram of intracellular regulatory signal transduction pathways for the (A) upregulated and (B) downregulated genes in AFR. Master regulators are indicated by pink rectangles, transcription factors (TFs) are purple rectangles, and green rectangles are intermediate molecules, which have been added to the network during the search for master regulators from selected transcription factors. The genes which encode for the master regulators are highlighted through the red dashed lines connecting the genes (blue rectangles) to their respective master regulators (pink rectangles). The intensity of the violet shadow around master regulators represents the logFC value of the downregulation of the genes encoding these master regulator molecules.

Next, we used these composite modules to identify MTRs,^16^ which are defined as factors that regulate gene expression of both upregulated and downregulated genes mediated by TFs through positive feedback loops. Our analysis identified 2 MTRs, MCF2 and GP6, in the upregulated and 15 MTRs in the (Figure 2A) downregulated (Figure 2B) genes. We note that >50% of MTRs that downregulate genes in AFR patients included immune-associated factors such as *IL-8*, *IL-1B, IFN-G (IFN-γ* ), and *CXCL8* through various disease-associated TFs such as BCL6, which has been shown to modulate inflammation.^30^

**Figure 3:**
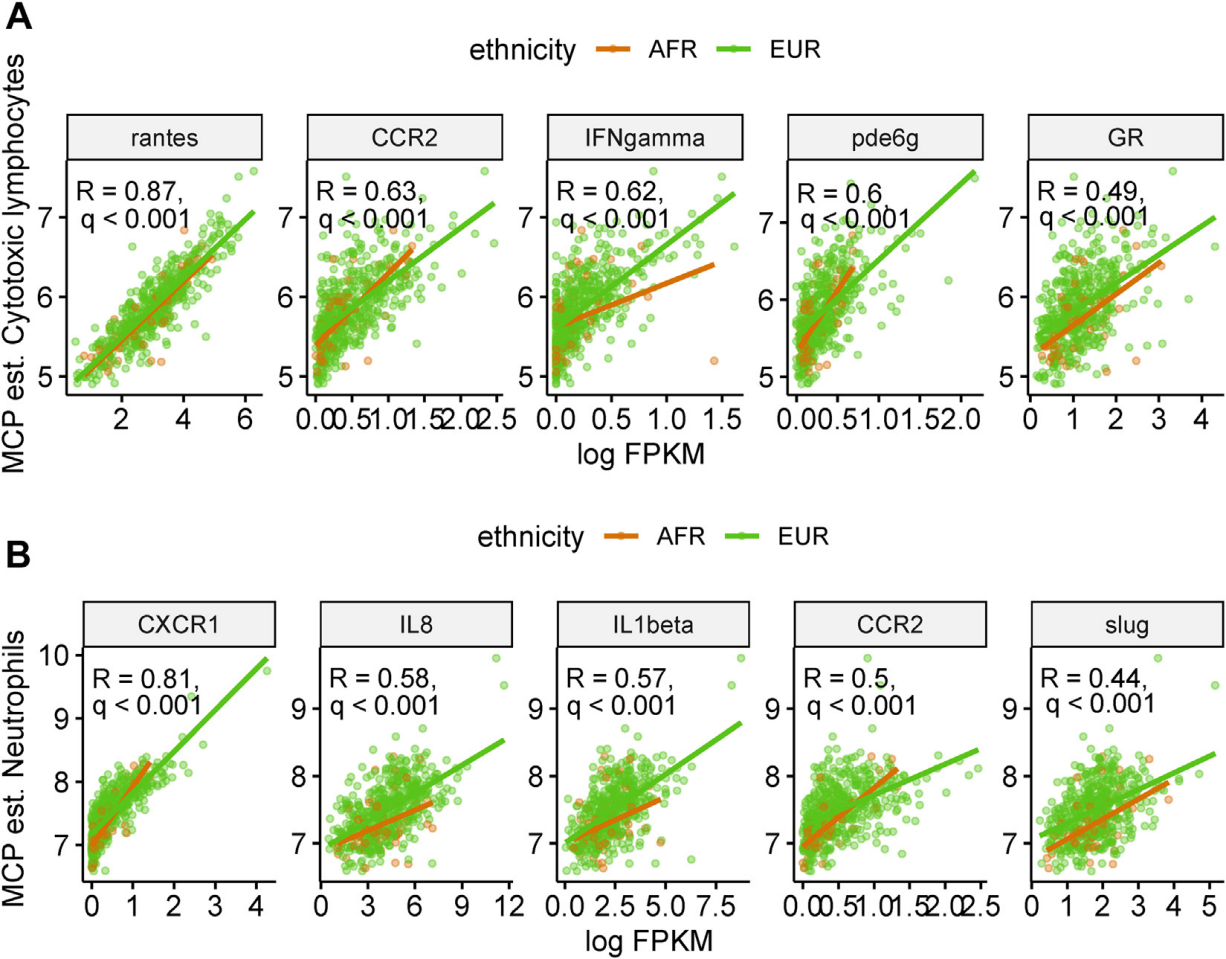
Correlation of a core set of MTRs and TFs with cytotoxic lymphocytes and neutrophil levels across both AFR and EUR patients with CRC. We carried out Pearson's correlation between log FPKM (x-axis) and cytotoxic lymphocyte and neutrophils estimate scores (as determined from the MCP-counter analysis) for all MTRs and TFs identified in our analysis. The five most significant correlations for cytotoxic lymphocytes and neutrophils are shown here: (A) correlation plots for log FPKM (x-axis) MTRs: *RANTES*, *CCR2*, *IFNgamma (IFN-γ)*, *PDE6G* (identified from downregulated genes), and TFs: glucocorticoid receptor (GR, identified from upregulated genes) and cytotoxic lymphocytes estimates (y-axis). (B) Correlation plots for log FPKM (x-axis) MTRs: *CXCR1, IL8, IL1β,* and *CCR2* (identified from downregulated genes) and TFs: SLUG (identified from upregulated genes) and neutrophil estimates (y-axis). The correlation value for each correlation analysis is displayed in individual plots, q < 0.001.

We subsequently sought to understand further the extent of regulatory impact of the MTRs and TFs on the cytotoxic lymphocyte and neutrophil levels in both AFR and EUR patients. To this purpose, we correlated the overall levels of cytotoxic lymphocytes and neutrophils with expression levels of the MTRs and TFs in AFR and EUR patients with CRC. Four MTRs (CCR2, PDE6G, RANTES, and *IFN-γ*) and one TF (GR) showed a significant positive correlation (Pearson’s correlation: 0.49–0.87, q < 0.001) with cytotoxic lymphocytes for both AFR and EUR patients with CRC (Figure 3A). Similarly, four MTRs (CCR2, CXCR1, IL1-B, and IL8) and one TF (SLUG) had a significant positive correlation (Pearson’s correlation: 0.44–0.8, q < 0.001) with neutrophils in both AFR and EUR patients with CRC (Figure 3B). These results strongly suggest that regulation of immune-associated genes is orchestrated by complex signaling pathways governed by MTRs and TFs, which ultimately contribute to the observed differences in the tumor microenvironment between AFR and EUR patients with CRC.

### Differential DNA methylation of specific disease-associated loci in AFR patients with CRC

**Figure 4:**
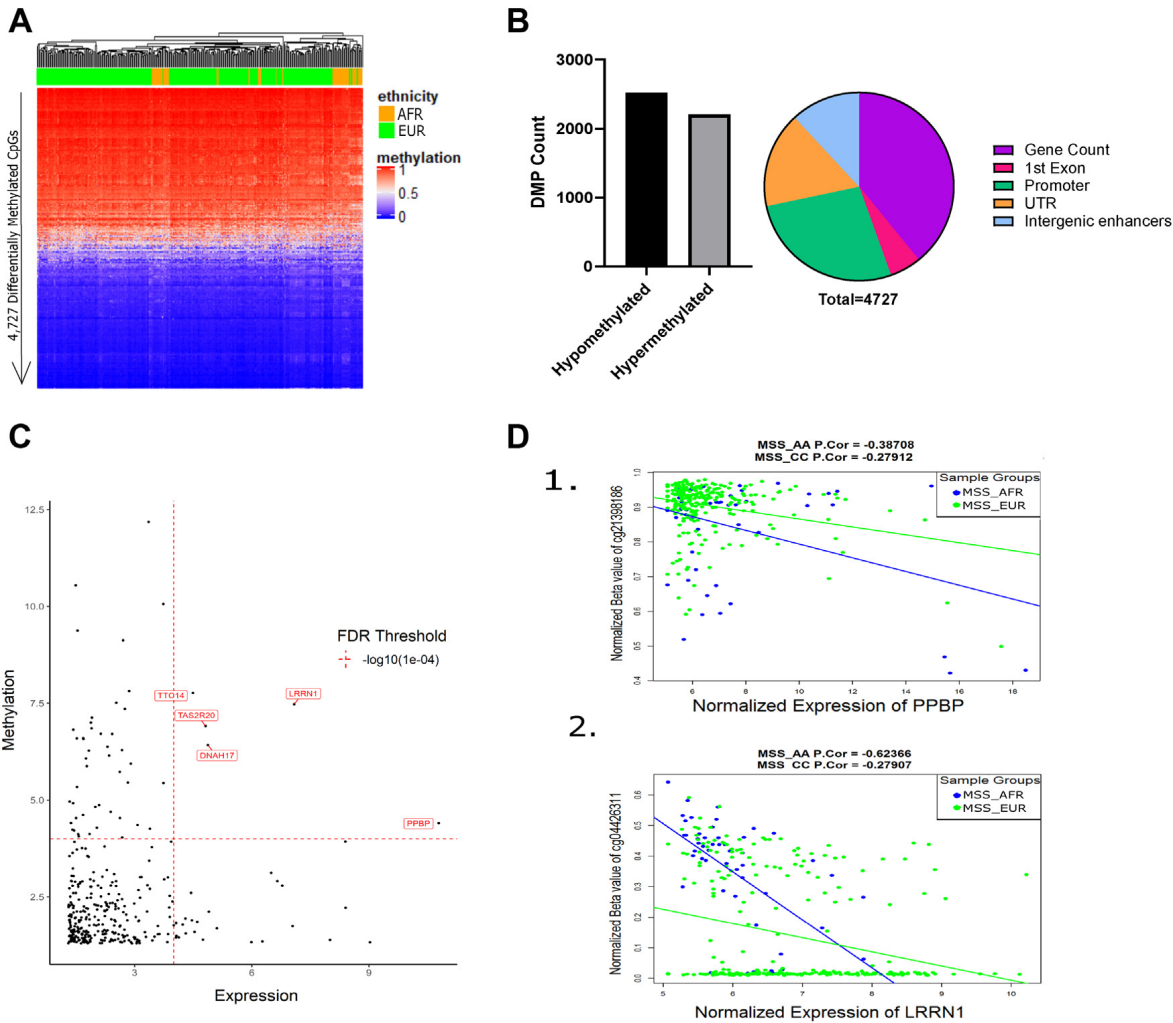
DNA methylation differences and identification of methylation-sensitive genes between AFR and EUR patients with CRC. (A) The heatmap was generated using the 4727 significantly (q < 0.05) tumor-specific DMPs between AFR and EUR patients with CRC. Unsupervised hierarchical clustering is denoted on the top of the heatmaps, and the ancestry of each patient is shown below the clusters in green (EUR) and yellow (AFR). Each row in the heatmap represents a single CpG site, and each column represents a patient. The color for each CpG site is based on the β-methylation value for the specific site ranging from 0 to 1, where 0 indicates 0% methylation, and 1 would indicate 100% methylation. (B) The bar plot shows the number of differentially methylated CpG positions (DMP) (y-axis) that are either significantly (q < 0.05) hypermethylated or hypomethylated (x-axis). The pie chart shows the distribution of the DMPs across various genomic features including gene body, first exon, promoter, both 5′ and 3′ untranslated regions (UTRs), and intergenic enhancers. (C) The plot shows which genes are both highly differentially expressed and methylated at the same time. The FDR threshold used in the plot indicates the value used in the plot for determining “high differential”. The x-axis is -log_10_(differentially expressed gene FDRs), and the y-axis shows the -log_10_(DMP FDRs). The genes with red color are differentially expressed and methylated having the FDR below 0.001. (D) These plots show correlation between the normalized β-methylation value (y-axis) for the CpG site associated with normalized gene expression (x-axis), specifically for genes whose expression is significantly inversely correlated with DNA methylation levels (q < 0.05) for at least one ancestry group. The association of the gene and CpG site was based on the presence of a CpG position either in the promoter (−1.2 Kb to +200 bp relative to the transcriptional start site) or 3’/5′-UTR or gene body. Correlation Pearson’s coefficient (Cor) and *P*-value for each gene are as follows: *PPBP*—AFR: Cor = −0.38, q = 0.004, EUR: Cor = −0.22, q = 0.0001; *LRRN1*—AFR: Cor = −0.63, q < 0.0001, EUR: Cor = −0.266, q < 0.0001.

Epigenetic alterations such as DNA methylation have been widely reported to regulate expression of key immune checkpoint proteins including PD-L1 and CTLA-4 in breast and colon cancer.^18^ To establish if DNA methylation changes also affect tumor microenvironment differences between AFR and EUR patients with CRC, we carried out differential DNA methylation analysis between the two patient cohorts. After subtraction of normal tissue-associated methylation (see methods) and correction for clinical covariates including age and tumor location, we identified 4727 tumor-specific DMPs representing methylation differences solely due to ancestry between AFR and EUR patients with CRC (Figure 4A). These DMPs comprised 2206 hypermethylated and 2522 hypomethylated DMPs in AFR relative to EUR patients (Figure 4A, B Table A4). Four hundred eighty-three of these DMPs were located in regions proximal to DEGs identified through differential expression analysis. Five of these genes—*TTO14, TASR20, DNAH17, PPBP,* and *LRRN1*—were found to be both strongly differentially expressed and differentially methylated (FDR < log_10_1e-04, Figure 4C). Further analysis revealed a significant inverse correlation (q < 0.001) between DNA methylation and gene expression levels for 2 of these 5 genes including *PPBP* (hypomethylated and upregulated) and *LRRN1* (hypermethylated and downregulated) across both AFR and EUR patients (Figure 4D, Figure A8). Notably, *PPBP* (also known as *CXCL7*) is a critical chemokine ligand that plays a critical role in promoting tumor proliferation through the CXCL7/CXCR1/2 signaling pathway.^31^ These results suggest that DNA methylation plays a role in regulating the expression of disease-associated genes that contribute to the progression of patients with CRC with AFR ancestry.

Our differential gene expression analysis suggests that certain TFs play a major role in mediating disease-associated expression alterations. One of the less explored mechanisms of TF-mediated gene expression changes involves the association of TFs with promoters and enhancers, resulting in changes in DNA methylation status of these regions and thus consequentially altering gene expression. To determine if this is the case in AFR patients with CRC, we identified TFBSs within hypermethylated or hypomethylated CpG sites in the vicinity (± 1 kb) of downregulated or upregulated genes, respectively. This analysis identified TFBSs for 94 TFs enriched at hypermethylated CpG sites associated with downregulated genes and 242 TFs enriched at hypomethylated CpG sites associated with upregulated genes. Of these, 52 TFBSs were found in both hypermethylated and hypomethylated sites, suggesting that binding of these TFs is independent of the level of methylation (hypermethylated or hypomethylated). However, 42 and 190 TFBSs were unique to hypermethylated and hypomethylated CpG positions, respectively. Furthermore, we observed that of the TFs identified to be involved in the MTR-mediated differential gene expression regulation (Figure 2A), TFBSs for only BCL6, RARG, and LEF1 were located within hypomethylated CpG sites associated with upregulated genes. The remaining TFs were either not enriched in this analysis or showed enrichment in both hypermethylated and hypomethylated CpG positions. In this context, binding of RARG and LEF1 has not been associated with direct epigenetic regulation. However, BCL6 has been shown to exhibit a high degree of cooperativity with chromatin-modifying molecules associated with promoter regions, resulting in epigenetic regulation of these loci.^32^ It is therefore likely that regulation of gene expression by MTRs via TFs, such as BCL6, is mediated by change of DNA methylation status of the TFBSs after binding of BCL6 to these sites.

## Discussion

AFR with CRC have overall worse prognosis, incidence, and mortality than EUR patients. Although reasons for these health disparities have been attributed partly to social determinants of health and long-standing effects of structural racism, the contribution of ancestry-associated molecular factors has not been thoroughly investigated.^10^ Recent reports suggest that differences in the tumor microenvironment could be a contributing factor to the overall difference in patient survival.^12^ However, the precise molecular processes that regulate these phenotypic differences remain largely unknown. Through a comprehensive multiomics analysis, our study elucidates an intricate interplay between MTRs, TFs, and DNA methylation that orchestrate the differences in immune cell infiltrates in the tumor microenvironment in AFR and EUR patients with CRC.

Decreased lymphocytic response in CRC tumors has been associated with worse survival,^15^ and we find that AFR with CRC have decreased cytotoxic lymphocytes and neutrophils compared with EUR patients. Although the reduced neutrophil levels in AFR patients with CRC may be related to the well-characterized benign ethnic neutropenia observed in AFR ancestry patients,^33^ our findings reveal a likely immune exhaustion phenomenon in AFR patients with CRC underpinned by reduced cytotoxic lymphocyte infiltrates in this patient population. This is consistent with a recent report examining the tumor microenvironment in African Americans with breast cancer, which demonstrates that African Americans with an exhausted CD8+ T cell signature present a more aggressive disease with poorer survival.^34^ Similarly, CD8+ T cells and cytotoxic T lymphocyte activity are reported to be reduced in late-stage CRC and are considered to be prognostic in nature.^29^^,^^35^ Although our results do not identify cytotoxic lymphocytes or neutrophils to be prognostic markers in the AFR patients with CRC (likely due to the small number of patients), however, our gene expression–based analysis corroborates previous immunohistochemistry-based findings showing that patients with CRC of AFR ancestry have lower levels of tumor-infiltrating lymphocytes relative to EUR patients.^15^

These differences in immune infiltrates between AFR and EUR patients can be explained largely by underlying gene expression alterations. We observe that immunological processes, such as cytokine-cytokine interactions, neutrophil chemotaxis, and chemokine activity, are significantly enriched in genes downregulated in the AFR patients with CRC. Prior reports evaluating gene expression and race in CRC show conflicting relationships between immune-related gene expression and race. Although some studies showed African Americans with CRC had higher expression of immune-related genes,^36^ others show that African Americans and Whites have similar compositions of CD8+ and CD57+ cells but lower levels of GzmB+ (granzyme B) staining which is a mediator of cytotoxicity.^37^^,^^38^

These immune-associated pathways are regulated by an intricate network of genetic and epigenetic factors. We are the first to provide evidence of a hierarchical signal transduction pathway governed by “MTRs” that downregulate expression of genes such as *IL-8*, *IL-1β*, *CXCR1 CXCL8,* and *Granzyme B*, which are central to eliciting an inflammatory response. This appears to be mediated by disease-associated TFs through a positive feedback loop. One such TF identified in our analysis is BCL6. Recent studies have shown that BCL6 plays a critical role as a modulator of immune response and inflammation and has been shown to exert its effect through regulation of genes such as *IL-6* and *Gata3* in macrophages and T-regulatory cells, respectively.^39^^,^^40^ This supports the hypothesis that TFs such as BCL6 likely play an important role in mediating the tumor microenvironment differences observed between AFR and 10.13039/501100001828EUR ancestry patients with CRC.

Cross-talk between inflammatory processes and tumor-infiltrating lymphocytes such as cytotoxic lymphocytes mediates the primary features of the overall tumor microenvironment across cancer types including CRC. It is highly likely that the regulatory impact of these key MTRs and TFs could be responsible for the differences in cytotoxic lymphocytes and neutrophil levels in AFR patients with CRC. Tellingly, a core set of these MTRs and TFs identified by our analysis exhibit a strong correlation with levels of cytotoxic lymphocytes and neutrophils across both AFR and EUR patients. These MTRs included molecules such as RANTES (also known as CCL5) and CCR2 (C-C motif chemokine receptor), with RANTES shown to downregulate expression of the *CCL5* gene in the AFR patients (Figure 2B). These results are consistent with previous findings that report of a strong correlation between CCL5 and neutrophil infiltration, which along with activated cytotoxic T lymphocytes drives active inflammatory processes in conditions such as chronic gastritis.^41^ Similarly, another MTR, IFN-γ, which showed significant correlation with neutrophils in our study and regulates expression of the *IFN-γ* gene itself, is shown to be primarily secreted by lymphocytes/dendritic cells in the colon and is significantly repressed in patients with CRC. In addition, high levels of IFN-γ in the serum from patients with CRC correlate with nonmetastatic CRC, further indicating its protective role as an antitumor molecule.^42^ More importantly, IFN-γ signaling promotes CD8+-mediated cytotoxicity.^43^^,^^44^ This evidence suggests that MTRs play an important role in the downregulation of immune and antitumor genes such as *CCL5* and *IFN-γ*, leading to reduced levels of cytotoxic lymphocytes and a more aggressive disease progression capacity in patients with CRC of AFR ancestry.

We also observe a significant positive correlation between two TFs, the glucocorticoid receptor and SLUG, with cytotoxic lymphocytes and neutrophils, respectively. Their binding sites are enriched in the upregulated genes in AFR CRC. Although there are no reports demonstrating a correlation between SLUG and cytotoxic lymphocytes and neutrophils, high expression of SLUG has been associated with poor recurrence-free survival in patients with gastrointestinal stromal tumor, potentially due to its prosurvival signaling properties in this tumor type.^45^ Moreover, signaling pathways regulated by TFs of the Snail family (that SLUG belongs to) induce the epithelial mesenchymal transition, thus accelerating metastasis and immunosuppression in melanoma cell lines.^46^ This mechanism may also occur in CRC. Unlike SLUG, the glucocorticoid receptor has been shown to transactivate dysfunctional gene expression, resulting in development of nonfunctional CD8+ tumor-infiltrating lymphocytes from naïve CD8+ cells.^47^ Taken together, these results suggest a dynamic role of specific MTRs and TFs that regulate aberrant gene expression in patients with CRC with AFR ancestry leading to a consequential loss of antitumor immunity.

Epigenetic modification such as DNA methylation and histone modifications also modulate cancer-associated phenotypes. Overall, we observe a trend toward tumor-specific hypermethylation at CpG sites in AFR compared with EUR ancestry patients with CRC. A recent study demonstrated that treatment of CD8+ cells with DNA hypomethylating agents promoted cytolytic activity of CD8+, resulting in suppression of tumor growth.^48^ Taking this recent report into consideration along with our findings showing increased DNA methylation levels and reduced cytotoxic lymphocyte abundance in patients with CRC with AFR ancestry, it suggests that DNA hypomethylating agents could represent a plausible therapeutic option to promote tumor suppressive immune mechanisms in patients with CRC of AFR ancestry. Furthermore, our integration analysis showed that differences in DNA methylation between AFR and EUR patients with CRC impact expression of immune mediators such as *PPBP* (also known as CXCL7).^31^^,^^49^ Taken together, our data suggest that dysregulated expression of *PPBP* through DNA methylation alterations provides a protumor survival advantage for AFR patients with CRC, and further studies are warranted to ascertain their precise role in CRC pathogenesis.

It is widely accepted that DNA methylation changes at TFBSs suppress TF binding and ultimately regulate gene expression. However, recent findings in this field now suggest that TF binding to regulatory regions alters DNA methylation, resulting in gene expression alterations.^50^^,^^51^ We observed a strong association between the TF—BCL6—with hypomethylated CpG positions that are proximal to upregulated genes. Interestingly, previous reports suggest a potential cooperativity between BCL6 and chromatin-modifying proteins such as EZH2, LSD1, and the NCOR/HDAC3 complex, suggesting that BCL6 may act as an epigenetic re-programming TF in cancers such as B-cell lymphoma.^32^ Our findings suggest that BCL6 may play a similar role in CRC; however, this altered methylation state is a cause or consequence of BCL6 needs to be experimentally ascertained.

We acknowledge that the small sample size of AFR patients used in this study and that the immune cell differences observed are based on gene expression analysis are indeed limitations. The former reflects the broader problem of the under-representation of minority patients included in clinical research and cancer clinical trials.^52^^,^^53^ Despite this, our study presents the largest group of AFR patients with CRC used to date for a multiomics comparative study. Nevertheless, should a larger independent cohort of patients with CRC with AFR and EUR ancestry with multiomics data become available, validation of our findings using such a data set will be of high importance. In addition, further histopathological analysis of the identified immune cell differences between patients with CRC of AFR and EUR ancestry will indeed confirm our hypothesis. Furthermore, it is likely that tumor heterogeneity will have an impact on the various molecular alterations identified in this study. Thus, future studies involving single-cell sequencing approaches would enable us to confirm the impact of intertumoral heterogeneity on the various regulatory factors identified in this study involved in regulating tumor microenvironment differences between patients with CRC of AFR and EUR ancestry.

**Figure 5:**
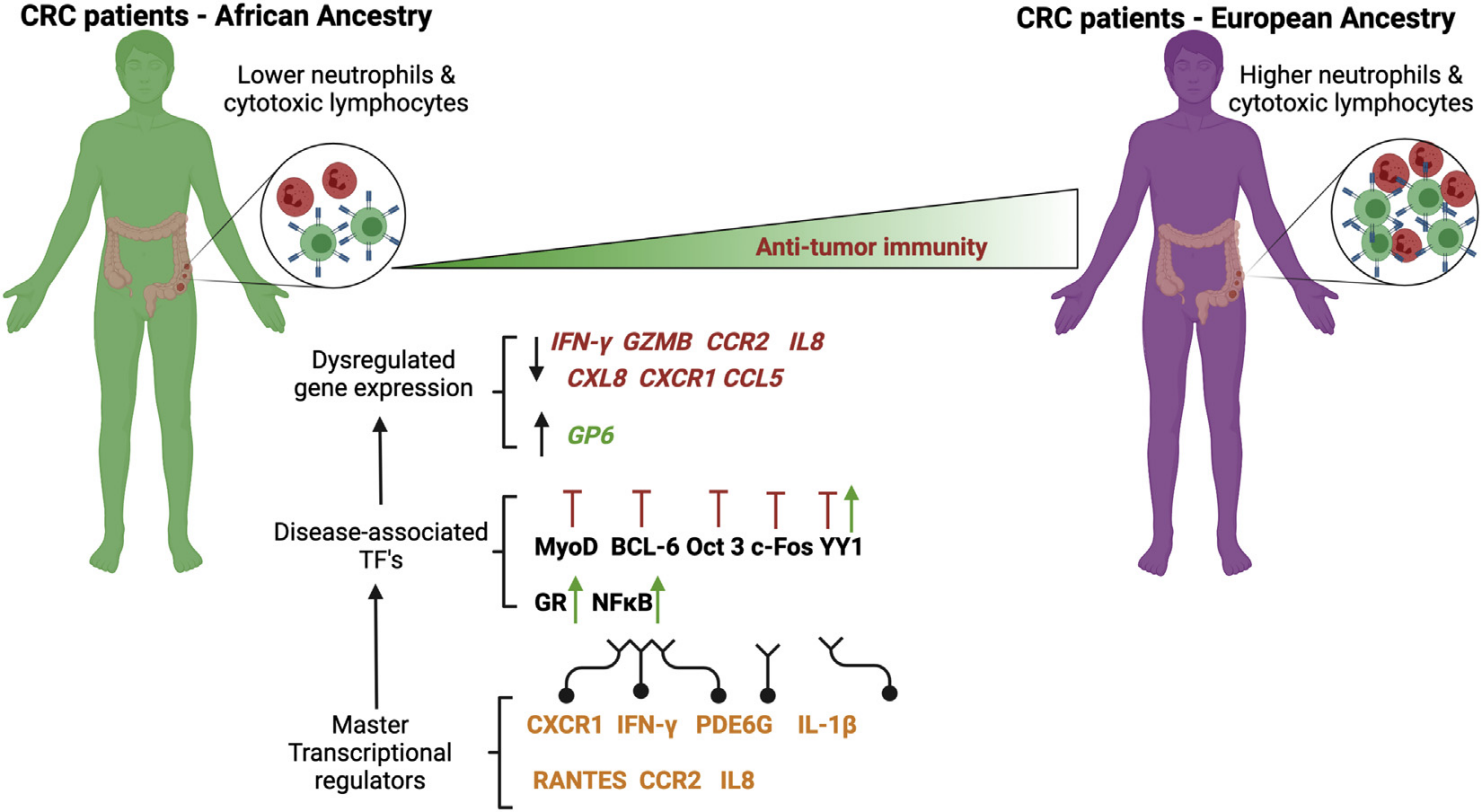
MTRs regulate expression of immunity-associated genes that orchestrate tumor microenvironment differences between AFR and EUR patients with CRC (*Figure created using**biorender.com*). The figure is an illustrative summary that demonstrates two key factors that affect tumor microenvironment differences between AFR and EUR patients with CRC, underpinned by a decreased antitumor immunity in AFR patients. We propose that master transcriptional regulators signaling through disease-associated TFs lead to dysregulated expression of several immunity-associated genes and genes involved in inhibiting tumor growth, resulting in decreased levels of neutrophils and cytotoxic lymphocytes in AFR patients with CRC. Green upward pointing arrows indicate a stimulatory or positive regulatory effect. Red inhibitory lines (⊥) indicate an inhibitory or negative regulatory effect.

In conclusion, we show that MTRs along with disease-associated TFs regulate expression of key immunity-associated genes. These processes underpin differences in tumor immune infiltrates between patients with CRC of AFR and EUR ancestry. Our study is the first to provide a molecular insight into the regulatory landscape that mediates the well-known tumor microenvironment differences between these two ancestry groups. Furthermore, we demonstrate that complex multifactorial events governed by alterations in DNA methylation and concomitant gene expression changes in specific disease-associated genes contribute to the observed disparity in antitumor immunity between AFR and EUR patients with CRC (Figure 5). These regulatory factors could potentially be used to improve personalized diagnostics/treatment for individuals with CRC based on ancestry. Although racial and socioeconomic factors are major determinants of CRC health disparities, further studies are needed to investigate how environmental stressors affect regulation of the overall tumor immune environment, either mediated through epigenetic mechanisms and through MTRs and TFs, similar to what has been reported in other cancer types.^54^

## Supplementary data

Table A1

Table A2

Table A3

Table A4

Figure A1

Figure A2

Figure A3

Figure A4

Figure A5

Figure A6

Figure A7

Figure A8
