# Supplementary figures and images for: Master Transcription Regulators and Transcription Factors Regulate Immune-Associated Differences Between Patients of African and European Ancestry With Colorectal Cancer
Source: Gastro Hep Adv. 2022 Mar 30;1(3):328–41. doi: 10.1016/j.gastha.2022.01.004 (PMC9151447; doi:10.1016/j.gastha.2022.01.004)

# Supplementary Figure 1

A

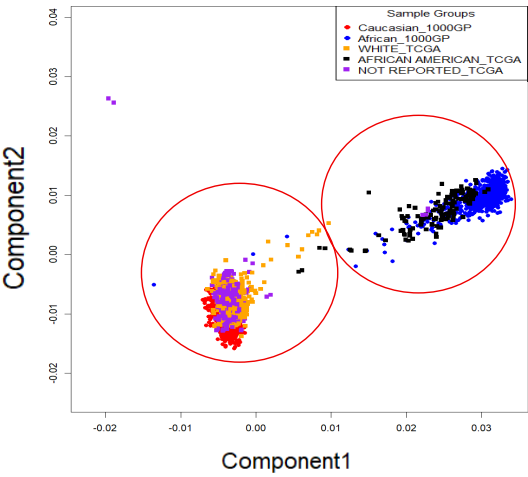

B

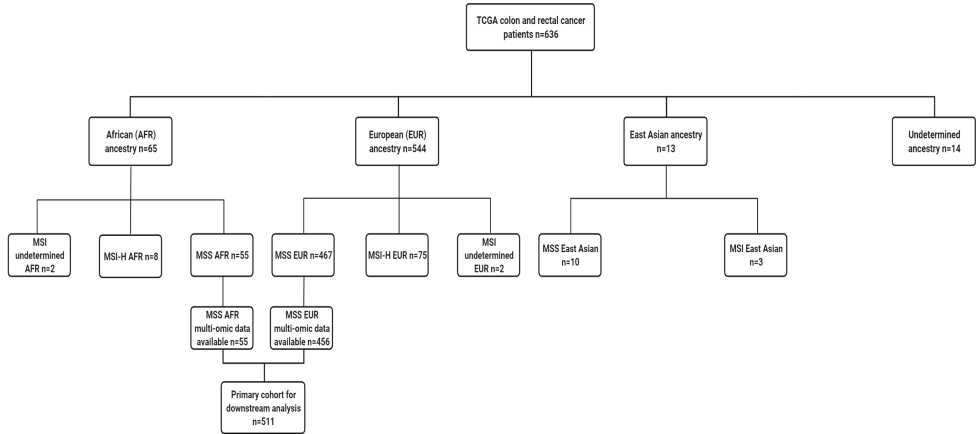

C

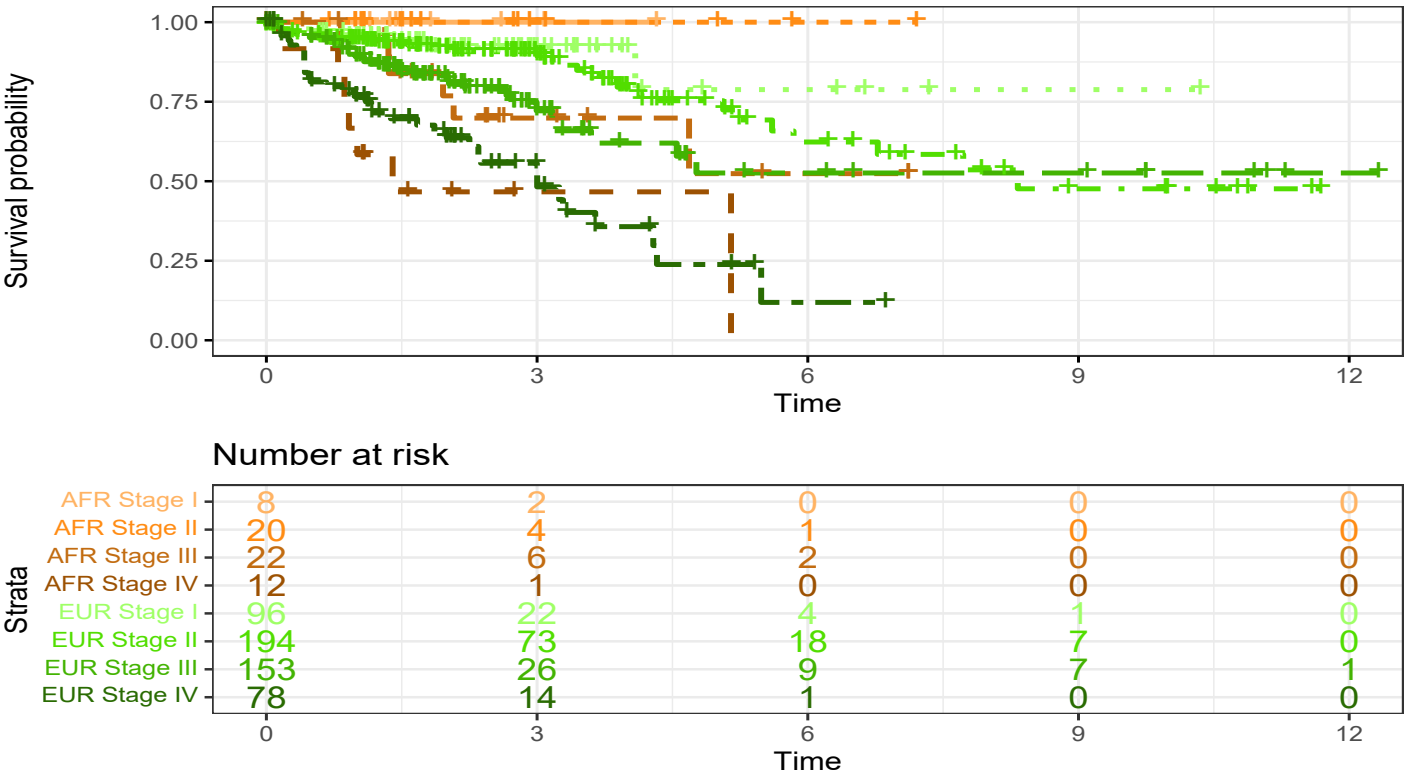

D

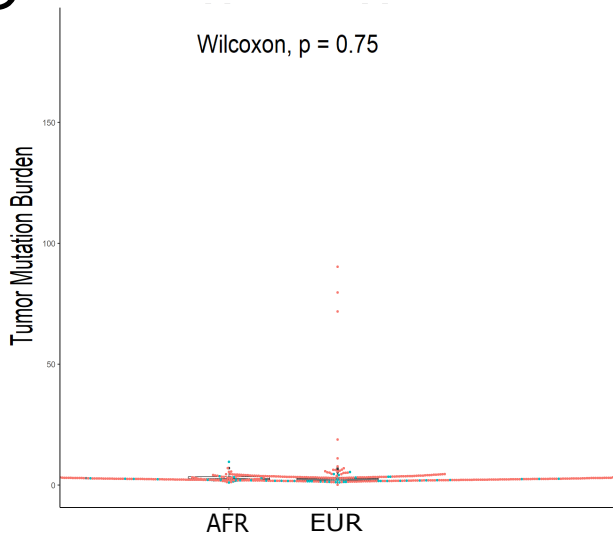

E

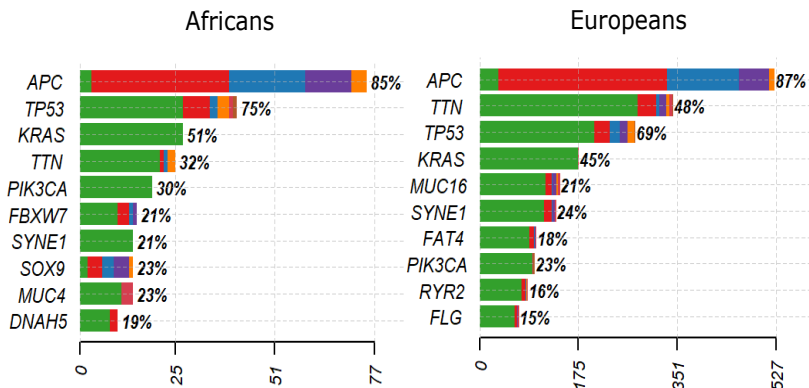

Supplement: Figure A1 [file mmc5.pdf]

# Supplementary Figure 2

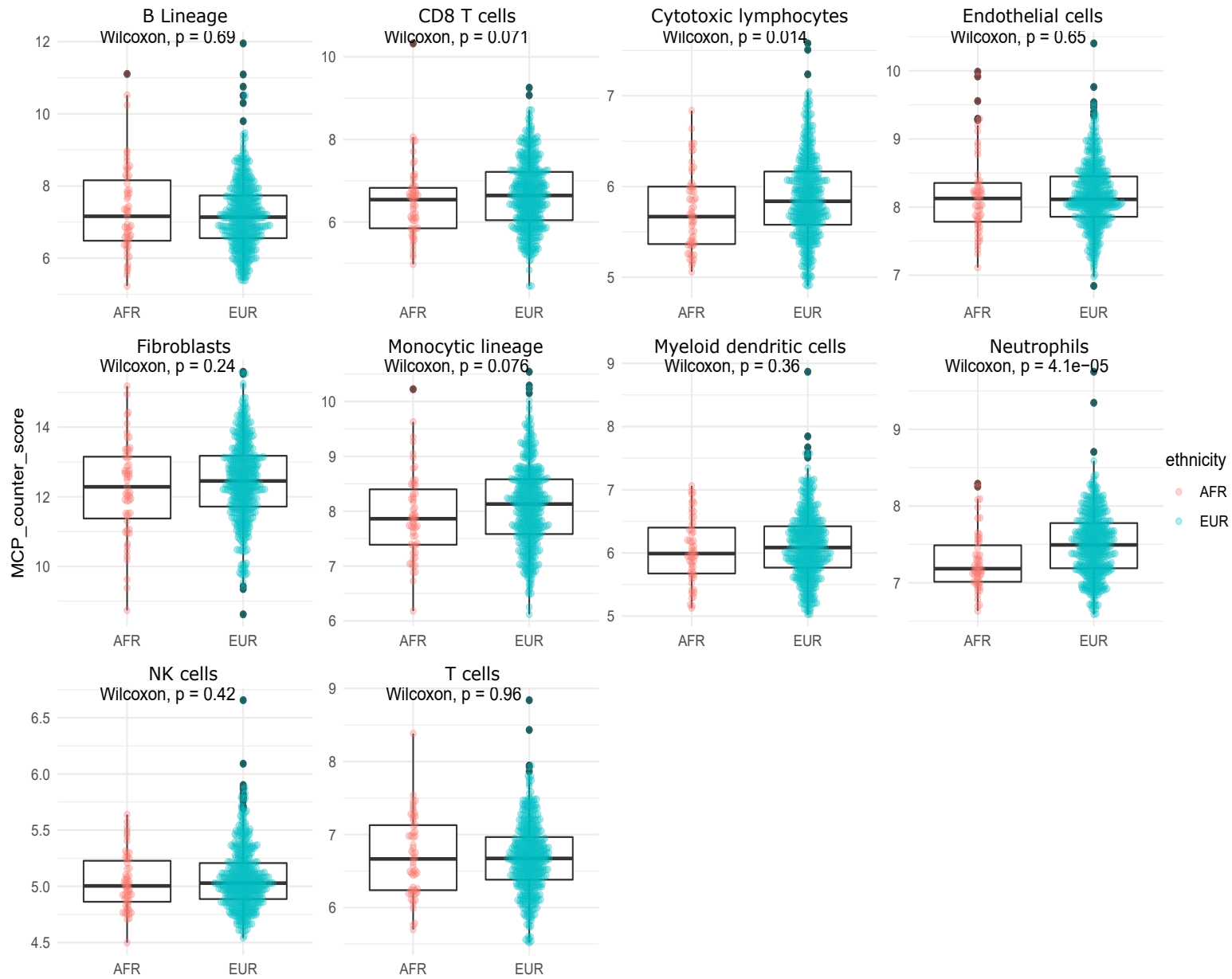

Supplement: Figure A2 [file mmc6.pdf]

# Supplementary Figure 3

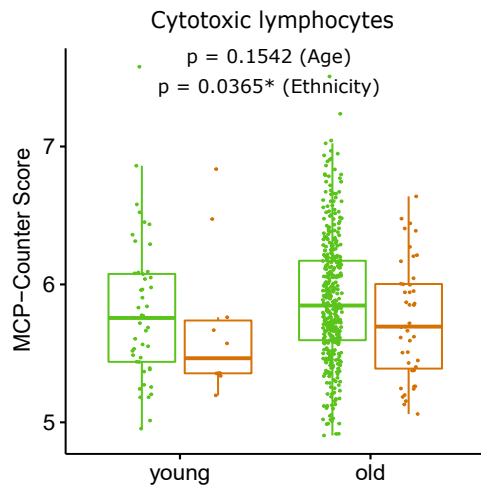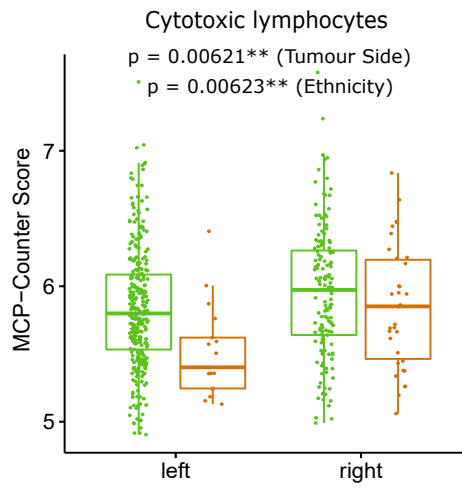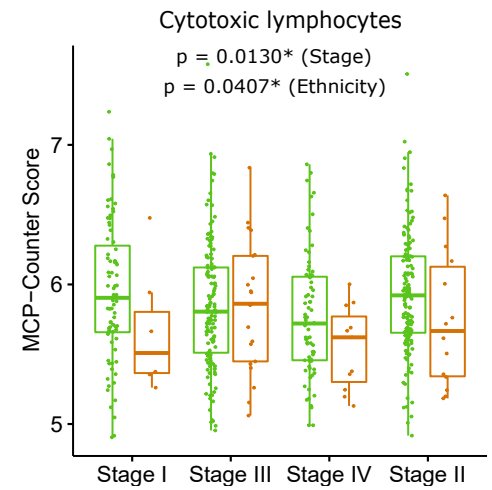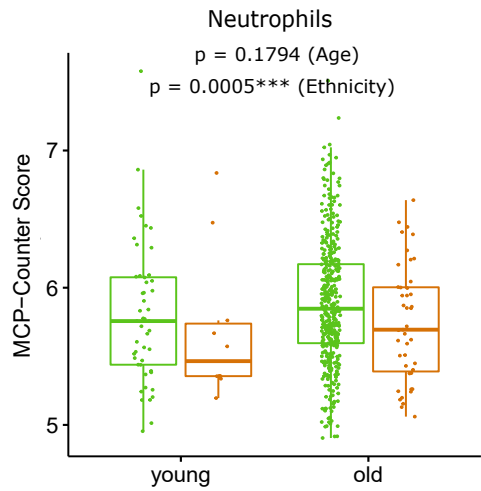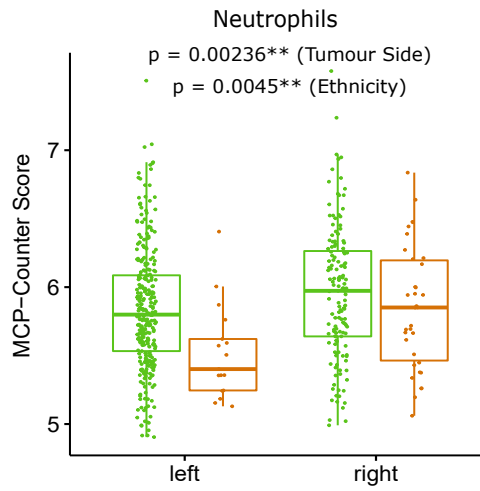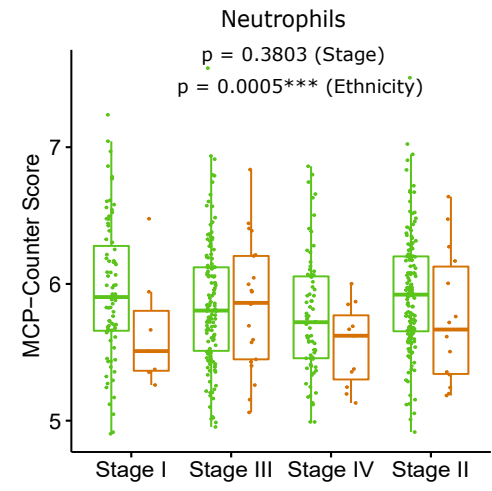

ethnicity 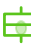 EUR 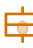 AFR

Supplement: Figure A3 [file mmc7.pdf]

# Supplementary Figure 4

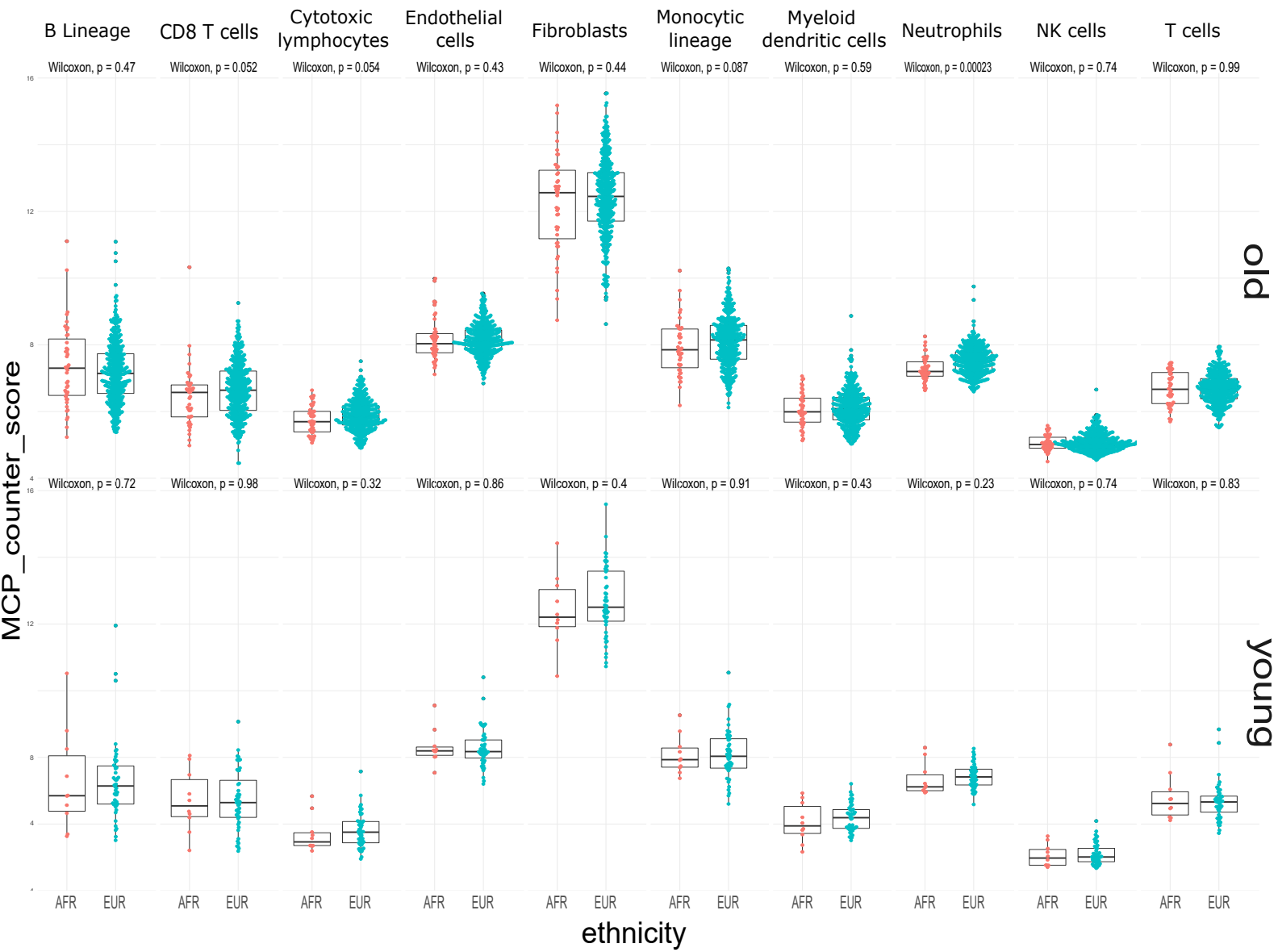

Supplement: Figure A4 [file mmc8.pdf]

# Supplementary Figure 5

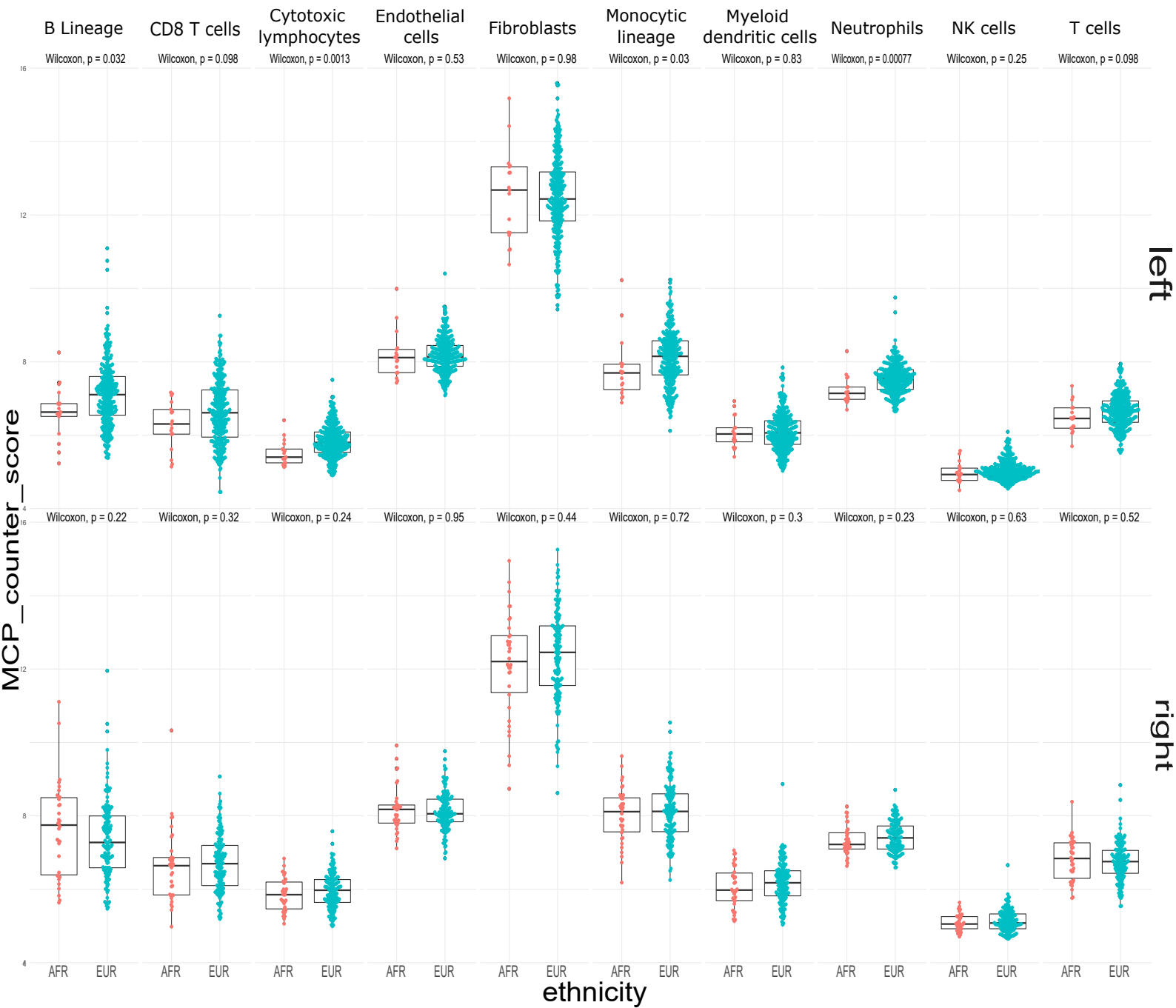

Supplement: Figure A5 [file mmc9.pdf]

# Supplementary Figure 6

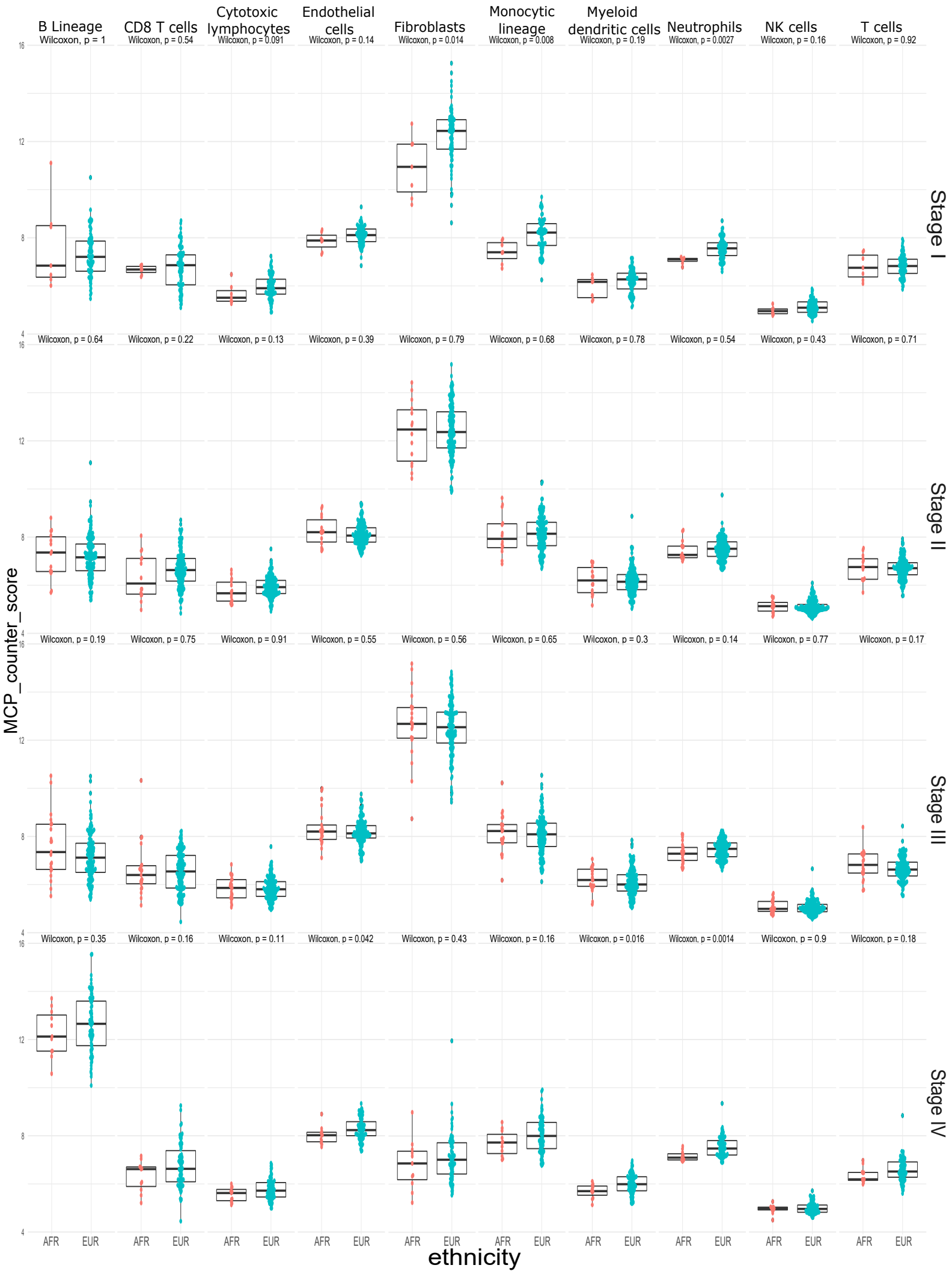

Supplement: Figure A6 [file mmc10.pdf]

Transcription Factor Binding Motifs

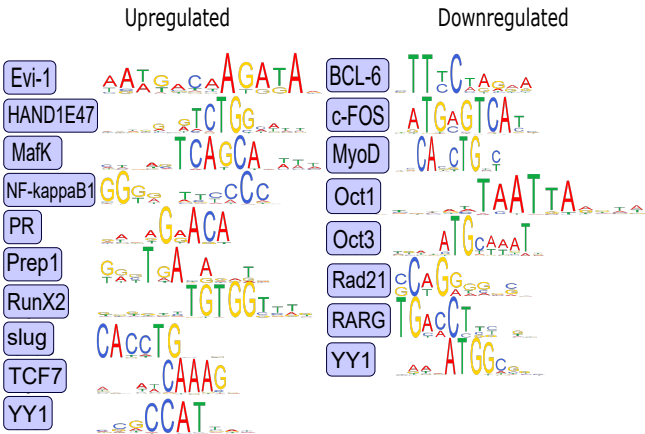

Supplement: Figure A7 [file mmc11.pdf]

# Supplementary Figure 8

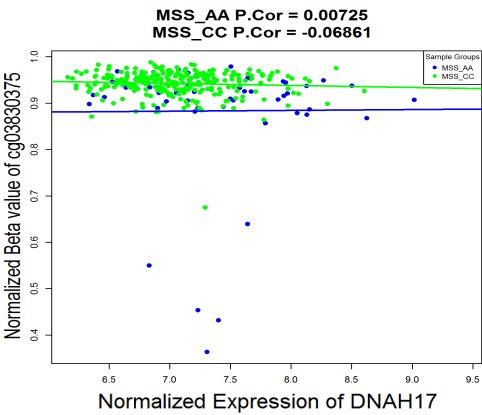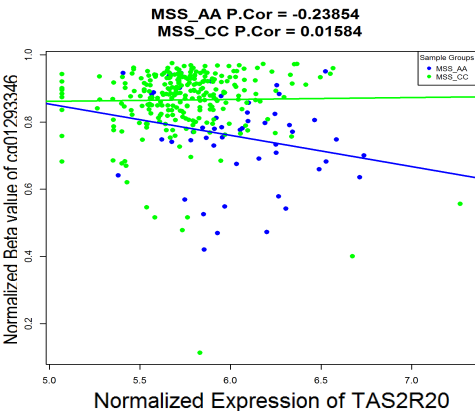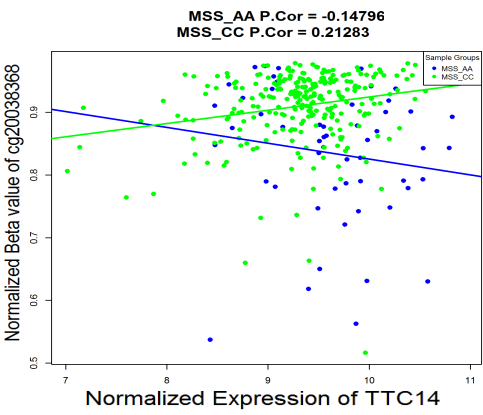

Supplement: Figure A8 [file mmc12.pdf]
